# Author Correction: Brain clocks capture diversity and disparities in aging and dementia across geographically diverse populations

**DOI:** 10.1038/s41591-024-03294-y

**Published:** 2024-09-16

**Authors:** Sebastian Moguilner, Sandra Baez, Hernan Hernandez, Joaquín Migeot, Agustina Legaz, Raul Gonzalez-Gomez, Francesca R. Farina, Pavel Prado, Jhosmary Cuadros, Enzo Tagliazucchi, Florencia Altschuler, Marcelo Adrián Maito, María E. Godoy, Josephine Cruzat, Pedro A. Valdes-Sosa, Francisco Lopera, John Fredy Ochoa-Gómez, Alfredis Gonzalez Hernandez, Jasmin Bonilla-Santos, Rodrigo A. Gonzalez-Montealegre, Renato Anghinah, Luís E. d’Almeida Manfrinati, Sol Fittipaldi, Vicente Medel, Daniela Olivares, Görsev G. Yener, Javier Escudero, Claudio Babiloni, Robert Whelan, Bahar Güntekin, Harun Yırıkoğulları, Hernando Santamaria-Garcia, Alberto Fernández Lucas, David Huepe, Gaetano Di Caterina, Marcio Soto-Añari, Agustina Birba, Agustin Sainz-Ballesteros, Carlos Coronel-Oliveros, Amanuel Yigezu, Eduar Herrera, Daniel Abasolo, Kerry Kilborn, Nicolás Rubido, Ruaridh A. Clark, Ruben Herzog, Deniz Yerlikaya, Kun Hu, Mario A. Parra, Pablo Reyes, Adolfo M. García, Diana L. Matallana, José Alberto Avila-Funes, Andrea Slachevsky, María I. Behrens, Nilton Custodio, Juan F. Cardona, Pablo Barttfeld, Ignacio L. Brusco, Martín A. Bruno, Ana L. Sosa Ortiz, Stefanie D. Pina-Escudero, Leonel T. Takada, Elisa Resende, Katherine L. Possin, Maira Okada de Oliveira, Alejandro Lopez-Valdes, Brian Lawlor, Ian H. Robertson, Kenneth S. Kosik, Claudia Duran-Aniotz, Victor Valcour, Jennifer S. Yokoyama, Bruce Miller, Agustin Ibanez

**Affiliations:** 1 Latin American Brain Health Institute, Universidad Adolfo Ibañez, Santiago de Chile, Chile; 2 Cognitive Neuroscience Center, Universidad de San Andrés, Buenos Aires, Argentina; 3 Department of Neurology, Massachusetts General Hospital and Harvard Medical School, Boston, MA USA; 4 Universidad de los Andes, Bogota, Colombia; 5 Global Brain Health Institute (GBHI), University of California, San Francisco, CA USA; 6 Global Brain Health Institute (GBHI), Trinity College Dublin, Dublin, Ireland; 7 The University of California Santa Barbara (UCSB), Santa Barbara, CA USA; 8 Escuela de Fonoaudiología, Universidad San Sebastián, Santiago de Chile, Chile; 9 Grupo de Bioingeniería, Decanato de Investigación, Universidad Nacional Experimental del Táchira, San Cristóbal, Venezuela; 10 Advanced Center for Electrical and Electronic Engineering, Universidad Técnica Federico Santa María, Valparaíso, Chile; 11 University of Buenos Aires, Buenos Aires, Argentina; 12 The Clinical Hospital of Chengdu Brain Sciences Institute, University of Electronic Sciences and Technology of China, Chengdu, China; 13 Technology of China, Chengdu, China; 14 Cuban Neuroscience Center, La Habana, Cuba; 15 Grupo de Neurociencias de Antioquia (GNA), University of Antioquia, Medellín, Colombia; 16 Department of Psychology, Master Program of Clinical Neuropsychology, Universidad Surcolombiana Neiva, Neiva, Colombia; 17 Department of Psychology, Universidad Cooperativa de Colombia, Arauca, Colombia; 18 Neurocognition and Psychophysiology Laboratory, Universidad Surcolombiana, Neiva, Colombia; 19 Reference Center of Behavioural Disturbances and Dementia, School of Medicine, University of Sao Paulo, Sao Paulo, Brazil; 20 Traumatic Brain Injury Cognitive Rehabilitation Out-Patient Center, University of Sao Paulo, Sao Paulo, Brazil; 21 Center for Social and Cognitive Neuroscience, School of Psychology, Universidad Adolfo Ibáñez, Santiago, Chile; 22 Neuropsychology and Clinical Neuroscience Laboratory (LANNEC), Physiopathology Program-Institute of Biomedical Sciences (ICBM), Neuroscience and East Neuroscience Departments, University of Chile, Santiago, Chile; 23 Centro de Neuropsicología Clínica (CNC), Santiago, Chile; 24 Faculty of Medicine, Izmir University of Economics, Izmir, Turkey; 25 Brain Dynamics Multidisciplinary Research Center, Dokuz Eylul University, Izmir, Turkey; 26 Izmir Biomedicine and Genome Center, Izmir, Turkey; 27 School of Engineering, Institute for Imaging, Data and Communications, University of Edinburgh, Edinburgh, UK; 28 Department of Physiology and Pharmacology ‘V. Erspamer’, Sapienza University of Rome, Rome, Italy; 29 Hospital San Raffaele Cassino, Cassino, Italy; 30 School of Psychology, Trinity College Dublin, Dublin, Ireland; 31 Department of Neurosciences, Health Sciences Institute, Istanbul Medipol University, İstanbul, Turkey; 32 Health Sciences and Technology Research Institute (SABITA), Istanbul Medipol University, Istanbul, Turkey; 33 Department of Biophysics, School of Medicine, Istanbul Medipol University, Istanbul, Turkey; 34 Pontificia Universidad Javeriana (PhD Program in Neuroscience), Bogotá, Colombia; 35 Center of Memory and Cognition Intellectus, Hospital Universitario San Ignacio Bogotá, San Ignacio, Colombia; 36 Departamento de Medicina Legal, Psiquiatría y Patología, Universidad Complutense de Madrid, Madrid, Spain; 37 Department of Electronic and Electrical Engineering, University of Strathclyde, Glasgow, UK; 38 Universidad Católica San Pablo, Arequipa, Peru; 39 Centro Interdisciplinario de Neurociencia de Valparaíso (CINV), Universidad de Valparaíso, Valparaíso, Chile; 40 Departamento de Estudios Psicológicos, Universidad ICESI, Cali, Colombia; 41 Centre for Biomedical Engineering, School of Mechanical Engineering Sciences, University of Surrey, Guildford, UK; 42 School of Psychology, University of Glasgow, Glasgow, UK; 43 Institute for Complex Systems and Mathematical Biology, University of Aberdeen, Aberdeen, UK; 44 Centre for Signal and Image Processing, Department of Electronic and Electrical Engineering, University of Strathclyde, Strathclyde, UK; 45 Sorbonne Université, Institut du Cerveau - Paris Brain Institute - ICM, InsermCNRS, Paris, France; 46 Department of Neurosciences, Health Sciences Institute, Dokuz Eylül University, Izmir, Turkey; 47 Harvard Medical School, Boston, MA USA; 48 Department of Psychological Sciences and Health, University of Strathclyde, Glasgow, UK; 49 BrainLat, Universidad Adolfo Ibáñez, Santiago, Chile; 50 Departamento de Lingüística y Literatura, Universidad de Santiago de Chile, Santiago, Chile; 51 Mental Health Department, Hospital Universitario Fundación Santa Fe, Bogota, Colombia; 52 Department of Geriatrics, Instituto Nacional de Ciencias Médicas y Nutrición Salvador Zubirán, Mexico City, Mexico; 53 Memory and Neuropsychiatric Center (CMYN), Neurology Department, Hospital del Salvador and Faculty of Medicine, University of Chile, Santiago, Chile; 54 Geroscience Center for Brain Health and Metabolism (GERO), Santiago, Chile; 55 Neuropsychology and Clinical Neuroscience Laboratory (LANNEC), Physiopathology Program – Institute of Biomedical Sciences (ICBM), Neuroscience and East Neuroscience Departments, University of Chile, Santiago, Chile; 56 Neurology and Psychiatry Department, Clínica Alemana-Universidad Desarrollo, Santiago, Chile; 57 Centro de Investigación Clínica Avanzada (CICA), Universidad de Chile, Santiago, Chile; 58 Departamento de Neurología y Neurocirugía, Hospital Clínico de la Universidad de Chile, Santiago, Chile; 59 Departamento de Neurociencia, Universidad de Chile, Santiago, Chile; 60 Servicio de Neurología, Instituto Peruano de Neurociencias, Lima, Perú; 61 Facultad de Psicología, Universidad del Valle, Cali, Colombia; 62 Cognitive Science Group, Instituto de Investigaciones Psicológicas (IIPsi), CONICET UNC, Universidad Nacional de Córdoba, Córdoba, Argentina; 63 Centro de Neuropsiquiatría y Neurología de la Conducta (CENECON), Universidad de Buenos Aires (UBA), Buenos Aires, Argentina; 64 Instituto de Ciencias Biomédicas (ICBM), Universidad Catoóica de Cuyo, San Juan, Argentina; 65 Instituto Nacional de Neurologia y Neurocirugia MVS, Universidad Nacional Autonoma de Mexico, Mexico City, Mexico; 66 Memory and Aging Center, Department of Neurology, Weill Institute for Neurosciences, University of California, San Francisco, CA USA; 67 Cognitive and Behavioral Neurology Unit, Hospital das Clinicas, University of São Paulo Medical School, São Paulo, Brazil; 68 Universidade Federal de Minas Gerais, Belo Horizonte, Brazil; 69 School of Engineering, Department of Electrical and Electronic Engineering, Trinity College Dublin, Dublin, Ireland; 70 Trinity College Institute of Neuroscience, Trinity College Dublin, Dublin, Ireland; 71 Trinity Centre for Biomedical Engineering, Trinity College Dublin, Dublin, Ireland; 72 Division of the Biological Sciences, The University of Chicago, Chicago, IL USA

**Keywords:** Cognitive ageing, Dementia, Developing world

Correction to: *Nature Medicine* 10.1038/s41591-024-03209-x, published online 26 August 2024.

In the version of the article initially published, Brian Lawlor’s name appeared incorrectly (as Brain) and has now been amended in the HTML and PDF versions of the article.

