# Supplementary material for: Brain clocks capture diversity and disparities in aging and dementia across geographically diverse populations
Source: Nat Med. 2024 Aug 26;30(12):3646–57. doi: 10.1038/s41591-024-03209-x (PMC11645278; doi:10.1038/s41591-024-03209-x)
Supplement: Supplementary file 1 — Supplementary Table 1 Results for gradient-boosting regressions with sex and gender inequality as predictors.Supplementary Table 2 Results for gradient-boosting regressions.Supplementary Table 3 Covariance analysis for years of education and age.Supplementary Table 4 fMRI acquisition.Supplementary Table 5 EEG acquisition.Supplementary Data 1 Details on fMRI and EEG data availability. [file 41591_2024_3209_MOESM1_ESM.pdf]

nature medicine

Supplementary information https://doi.org/10.1038/s41591-024-03209-x

Brain clocks capture diversity and disparities in aging and dementia across geographically diverse populations

In the format provided by the authors and unedited

Supplementary Information

Supplementary Table 1. Results for gradient boosting regressions with sex and gender inequality as predictors. We conducted a two-sided F-test to evaluate the overall significance of the regression model.

R^2 = 0.40 (99% CI ±0.12), F^2 = 0.66 (99% CI ±0.14), RMSE = 6.85 (99% CI ±0.82), F = 352.54, p < 1e-15						
Predictors	Mean Shaps	CI [99%]	Mean MDI	CI [99%]	Permutation Importance	CI [99%]
NCD	4.484	[4.107-4.861]	0.738	[0.704-0.772]	0.769	[0.762-0.776]
GII	0.894	[0.825-0.963]	0.048	[0.041-0.055]	0.158	[0.153-0.163]
Region	0.629	[0.56-0.697]	0.018	[0.014-0.022]	0.034	[0.031-0.037]
Sex	0.601	[0.527-0.68]	0.027	[0.022-0.032]	0.039	[0.037-0.041]

Note: NCD = neurocognitive disorders (HC vs. MCI, AD or bvFTD), GII = The Gender Inequality index, Region = Latin-American and Caribbean countries (LAC) vs non-LAC.

Supplementary Table 2. Results for gradient boosting regressions. We conducted a two-sided F-test to evaluate the overall significance of the regression model.

A. Models with the total dataset: R^2 = 0.415 (99% CI ±0.12), F^2 = 0.71 (99% CI ±0.14), RMSE = 6.755 (99% CI ±0.89), F = 253.39, p < 1e-15						
Predictors	Mean SHAPS	CI [99%]	Mean MDI	CI [99%]	Permutation Importance	CI [99%]
NCD	4.234	[3.847-4.621]	0.743	[0.712-0.774]	0.753	[0.746-0.76]
GINI	1.241	[1.15-1.332]	0.081	[0.073-0.089]	0.157	[0.15-0.164]
Pollution	0.386	[0.339-0.433]	0.013	[0.009-0.017]	0.037	[0.034-0.04]
NCDs	0.219	[0.181-0.257]	0.005	[0.003-0.007]	0.029	[0.027-0.031]
CMMN	0.178	[0.147-0.209]	0.004	[0.002-0.006]	0.024	[0.022-0.026]
B. Models with the LAC dataset: R^2 = 0.37 (99% CI ±0.17), F^2 = 0.588 (99% CI ±0.21), RMSE = 6.9 (99% CI ±0.92), F = 138.78, p < 1e-15						
Predictors	Mean SHAPS	CI [99%]	Mean MDI	CI [99%]	Permutation Importance	CI [99%]
NCD	4.021	[3.56-4.482]	0.739	[0.697-0.781]	0.751	[0.743-0.759]
GINI	1.378	[1.24-1.519]	0.11	[0.097-0.123]	0.157	[0.15-0.164]
CMMN	0.287	[0.235-0.34]	0.006	[0.003-0.009]	0.024	[0.022-0.026]
Pollution	0.284	[0.25-0.32]	0.018	[0.012-0.024]	0.042	[0.039-0.045]
NCDs	0.257	[0.21-0.305]	0.005	[0.003-0.007]	0.026	[0.024-0.028]
C. Models with the non-LAC dataset: R^2 = 0.41 (99% CI ±0.17), F^2 = 0.71 (99% CI ±0.21), RMSE = 6.6 (99% CI ±1.31), F = 135.91, p < 1e-15						
Predictors	Mean SHAPS	CI [99%]	Mean MDI	CI [99%]	Permutation Importance	CI [99%]
NCD	4.983	[4.536-5.43]	0.87	[0.821-0.919]	0.963	[0.959-0.967]
NCDs	0.246	[0.16-0.33]	0.004	[0.001-0.007]	0.01	[0.006-0.014]
Pollution	0.231	[0.15-0.308]	0.006	[0.002-0.01]	0.016	[0.012-0.02]
GINI	0.184	[0.111-0.257]	0.004	[0.002-0.006]	0.009	[0.005-0.013]
CMMN	0.063	[0.034-0.092]	0.001	[0.0-0.002]	0.002	[0.001-0.003]

Note: NCD = neurocognitive disorders (HC vs. MCI, AD or bvFTD), NCDs = non-communicable diseases, CMMN = communicable diseases.

Supplementary Table 3. Covariance analysis for years of education and age. We conducted an ANOVA analysis using a Type II sum of squares approach to evaluate the significance of predictors.

Comparisons	A. HC LAC vs. HC non-LAC				B. AD LAC vs. AD non-LAC			
Predictors	Sum squares	df	F	p value	Sum squares	df	F	p value
Group	601.0433	1	15.32718	0.000104	2427.091	1	54.61552	<1e-15
Education	31.76629	1	0.810071	0.368569	8.825269	1	0.19859	0.655959
Age	145.4403	1	3.708868	0.054739	64.49888	1	1.451384	0.228594
Residual	18077.75	461			43995.18	990		
Comparisons	C. HC non-LAC vs. AD non-LAC				D. HC non-LAC vs. MCI non-LAC			
Predictors	Sum squares	df	F	p value	Sum squares	df	F	p value
Group	22222.13	1	583.1485	<1e-15	9584.242	1	291.6454	<1e-15
Education	14.52674	1	0.381208	0.537166	19.72451	1	0.600211	0.438886
Age	83.90817	1	2.2019	0.138307	8.202627	1	0.249603	0.617587
Residual	25760.44	676			15511.17	472		
Comparisons	E. HC non-LAC vs. bvFTD non-LAC				F. HC LAC vs. AD LAC			
Predictors	Sum squares	df	F	p value	Sum squares	df	F	p value
Group	10778.54	1	298.5478	<1e-15	22427.48	1	477.9453	<1e-15
Education	8.502628	1	0.235509	0.627813	23.13814	1	0.49309	0.482763
Age	701.7014	1	19.43598	1.44E-05	73.81414	1	1.573031	0.210146
Residual	11155.89	309			36366.71	775		
Comparisons	G. HC LAC vs. MCI LAC				H. HC LAC vs. bvFTD LAC			
Predictors	Sum squares	df	F	p value	Sum squares	df	F	p value
Group	1103.314	1	23.05894	2.49e-06	14950.29	1	299.525	<1e-15
Education	3.553817	1	0.074274	0.785401	15.30054	1	0.306542	0.580148
Age	20.86055	1	0.435979	0.509578	15.30196	1	0.306571	0.58013
Residual	14306.42	299			18268.27	366		
Comparisons	I. MCI non-LAC versus AD non-LAC				J. MCI LAC versus AD LAC			
Predictors	Sum squares	df	F	p value	Sum squares	df	F	p value
Group	2450.071	1	60.16342	2.9e-14	2076.59	1	45.74226	3.4e-11
Education	0.175965	1	0.004321	0.947607	11.54885	1	0.254393	0.614197
Age	135.9108	1	3.337398	0.068124	2.203579	1	0.04854	0.825705
Residual	30135.46	740			25422.67	560		

Supplementary Table 4. Resting-state fMRI acquisition parameters per center and scanner

Location	Scanner Model Tesla	TR (ms)	TE (ms)	Voxel size (mm)	N° vol	Matrix dimension	N° slices	Duration (min)
AR Center A	Philips Ingenia 3T	2640	30	3x3x3	220	80x80	49	10
AR Center B	Philips Achieva 1.5T	2780	30	3.6x3.6x4	209	64x64	33	10
AR Center C	GE Signa Explorer 1.5T	2500	30	3.7x3.7x5	120	64x64	33	5
CH Center A	Siemens Skyra 3T	2660	30	3x3x3	300	76x76	46	10
CH Center B	Siemens Skyra 3T	2660	30	3x3x3	240	76x76	46	10
CH Center D	Siemens Avanto 1.5T	3300	50	3.5X3.5X3.7	190	64X64	36	10
CH Center I	Philips Ingenia 3T	4970	30	2.5x2.5x2.7	121	96x96	45	10
CH Center J	Philips Ingenia 3T	4970	30	2.5x2.5x2.7	121	96x96	45	10
CO Center A	Philips Achieva 3T	4000	30	2.8x2.8x3	150	80x80	40	10
CO Center B	Philips Achieva 3T	3000	30	3.3x3.3x3.3	197	64x64	48	10
CO Center C	Philips Achieva 3T	3000	30	3x3x3	160	80x80	40	8
CO Center D	Siemens 3T	2510	30	3x3x3	240	70x70	45	8
MX Center D	Philips Achieva 1.5T	3500	32	2.2x2.2x2.7	120	94x94	66	7
US Center 1	Philips Achieva 3T	3000	30	2.5x2.5x3.3	197	96x96	47	10
US Center 2	Philips Achieva 3T	3000	30	3.3x3.3x3.3	140	64X64	48	7
US Center 3	Philips Achieva 3T	3000	30	3.3x3.3x3.3	197	64X64	48	10
US Center 4	Philips Achieva 3T	3000	30	3.3x3.3x3.3	197	64X64	48	10
US Center 5	Philips Achieva 3T	3000	30	3.3x3.3x3.3	197	64X64	48	10
US Center 6	Philips Achieva dStream 3T	3000	30	3.3x3.3x3.3	197	64X64	48	10
US Center 7	Philips Achieva dStream 3T	3000	30	3.3x3.3x3.3	197	64X64	48	10
US Center 8	Siemens Biograph mMR 3T	3000	30	3.4x3.4x3.4	197	64X64	48	10
US Center 9	GE Discovery MR750 3T	3000	30	3.4x3.4x3.4	200	64X64	48	10
US Center 10	GE Discovery MR750 3T	3000	30	3.4x3.4x3.4	200	64X64	48	10
US Center 11	GE Discovery MR750 3T	3000	30	3.4x3.4x3.4	200	64X64	48	10
US Center 12	GE Discovery MR750 3T	3000	30	3.4x3.4x3.4	200	64X64	48	10
US Center 14	GE Discovery MR750 3T	3000	30	3.4x3.4x3.4	200	64X64	48	10
US Center 16	GE Discovery MR750 3T	3000	30	3.4x3.4x3.4	200	64X64	48	10
US Center 17	GE Discovery MR750 3T	3000	30	3.4x3.4x3.4	200	64X64	48	10
US Center 18	GE Discovery MR750 3T	3000	30	3.4x3.4x3.4	200	64X64	48	10

US Center 19	Philips Ingenia 3T	3000	30	3.4x3.4x3.4	197	64X64	48	10
US Center 20	Philips Ingenia 3T	3000	30	3.4x3.4x3.4	197	64X64	48	10
US Center 21	Philips Ingenia 3T	3000	30	3.4x3.4x3.4	197	64X64	48	10
US Center 22	Philips Ingenia 3T	3000	30	3.4x3.4x3.4	197	64X64	48	10
US Center 23	Philips Ingenia 3T	3000	30	3.3x3.3x3.3	140	64X64	48	7
US Center 24	Philips Ingenia 3T	3000	30	3.4x3.4x3.4	197	64X64	48	10
US Center 25	Siemens Prisma 3T	607	32	2.5x2.5x2.5	976	88x88	64	10
US Center 26	Siemens Prisma 3T	607	32	2.5x2.5x2.5	976	88x88	64	10
US Center 27	Siemens Prisma 3T	607	32	2.5x2.5x2.5	976	88x88	64	10
US Center 28	Siemens Prisma 3T	3000	30	3.4x3.4x3.4	197	64X64	48	10
US Center 29	Siemens Prisma 3T	3000	30	3.4x3.4x3.4	197	64X64	48	10
US Center 30	Siemens Prisma 3T	607	32	2.5x2.5x2.5	976	88x88	64	10
US Center 31	Siemens Prisma 3T	3000	30	3.4x3.4x3.4	197	64X64	48	10
US Center 32	Siemens Prisma 3T	3000	30	3.4x3.4x3.4	197	64X64	48	10
US Center 33	Siemens Prisma_Fit 3T	3000	30	3.4x3.4x3.4	197	64X64	48	10
US Center 34	Siemens Prisma_Fit 3T	607	32	2.5x2.5x2.5	976	88x88	64	10
US Center 35	Siemens Prisma_Fit 3T	3000	30	3.4x3.4x3.4	197	64X64	48	10
US Center 36	Siemens Prisma_Fit 3T	3000	30	3.4x3.4x3.4	197	64X64	48	10
US Center 37	Siemens Prisma_Fit 3T	3000	30	3.4x3.4x3.4	197	64X64	48	10
US Center 38	Siemens Prisma_Fit 3T	3000	30	3.4x3.4x3.4	197	64X64	48	10
US Center 39	Siemens Prisma_Fit 3T	607	32	2.5x2.5x2.5	976	88x88	64	10
US Center 40	Siemens Prisma_Fit 3T	607	32	2.5x2.5x2.5	976	88x88	64	10
US Center 41	Siemens Prisma_Fit 3T	607	32	2.5x2.5x2.5	976	88x88	64	10
US Center 42	Siemens Prisma_Fit 3T	3000	30	3.4x3.4x3.4	197	64X64	48	10
US Center 43	Siemens Prisma_Fit 3T	3000	30	3.4x3.4x3.4	197	64X64	48	10
US Center 44	Siemens Prisma_Fit 3T	607	32	2.5x2.5x2.5	976	88x88	64	10
US Center 45	Siemens Prisma_Fit 3T	3000	30	3.4x3.4x3.4	197	64X64	48	10
US Center 46	Siemens Prisma_Fit 3T	3000	30	3.4x3.4x3.4	197	64X64	48	10
US Center 47	GE Signa HDxt 3T	2925	30	3.3x3.3x3.3	160	64X64	48	8
US Center 48	GE Signa HDxt 3T	3000	30	3.4x3.4x3.4	200	64X64	48	10
US Center 49	GE Signa HDxt 3T	3000	30	3.3x3.3x3.3	200	64X64	48	10
US Center 50	GE Signa HDxt 3T	3000	30	3.3x3.3x3.3	200	64X64	48	10

US Center 51	Siemens Skyra 3T	3000	30	3.4x3.4x3.4	197	64X64	48	10
US Center 52	Siemens Skyra 3T	788	33	2.7x2.7x2.5	750	88x88	64	10
US Center 53	Siemens TrioTim 3T	3000	30	3.4x3.4x3.4	197	64X64	48	10
US Center 54	Siemens TrioTim 3T	3000	30	3.4x3.4x3.4	197	64X64	48	10
US Center 55	Siemens TrioTim 3T	3000	30	3.4x3.4x3.4	197	64X64	48	10
US Center 56	Siemens TrioTim 3T	3000	30	3.4x3.4x3.4	197	64X64	48	10
US Center 57	Siemens Verio 3T	3000	30	3.4x3.4x3.4	197	64X64	48	10
US Center 58	Siemens Verio 3T	3000	30	3.4x3.4x3.4	197	64X64	48	10
US Center 60	Siemens Verio 3T	3000	30	3.4x3.4x3.4	197	64X64	43	10
US Center 61	Siemens Verio 3T	3000	30	3.4x3.4x3.4	197	64X64	48	10
US Center 62	Siemens TrioTim 3T	2000	27	2.5x2.5x3.6	240	92x92	36	8
US Center 63	Siemens TrioTim 3T	2000	27	2.5x2.5x3.6	240	92x92	36	8
US Center 64	GE Signa HDxt 3T	2770	27	1.8x1.8x3.6	240	128x128	36	11
US Center 65	GE Discovery MR750 3T	2770	27	1.8x1.8x3.6	240	128x128	46	11
US Center 66	Siemens Prisma_Fit 3T	8500	32	2.2x2.2x2.2	560	96x96	66	8
US Center 67	Siemens TrioTim 3T	2000	27	2.5x2.5x3.5	240	92x92	36	8
US Center 68	Siemens TrioTim 3T	2000	27	2.5x2.5x3.5	240	92x92	36	8

AR: Argentina, CH: Chile, CO: Colombia, MX: Mexico, TE: echo time, TR: repetition time, US: United States.

Supplementary Table 5. EEG acquisition parameters

Center	Time	Eyes	Equipment	Channels	Ref.	Filter	Sampling rate
Centro de Neurociencias de Cuba (2)	2-20 min	Closed	Digital Electroencephalogra system MEDICID	64/128 Passive	Linked earlobe:	0.5-50 Hz	200 Hz
BrainLat (3,4)	5-25 min	Closed	Biosemi ADBOX-MODEL	128 Active	Mastoids	0.16-100 Hz	2048 Hz
Izmir University of Economics	7-10 min	Closed 4 mi Open 4 min	BrainAmp	32 Passive	A1 + A2 Electrodes	0.03-70 Hz	500 Hz
Trinity College Dublin	3-12 min	Closed	Two Biosemi system	264+7 sensors Active	Average	0.1-95 Hz	512 Hz
Universidad de Antioquia	5-8 min	Closed	Neuroscan Synamps 2	72 Passive	Vertex	0.1-200 Hz	200 Hz
	7-18 min	Closed	Neuroscan Synamps 2	72 Passive	Right mastoid	0.1-200 Hz	1024 Hz
Universidad de Sao (5)	10-26 min	Closed	Braintech 3.0	21 Passive	unknown	1-60 Hz	200 Hz
Sapienza Università di Roma	8-14 min	Closed 5 mi Open 5 min	Brain Amp	61 Passive	Fcz Electrode	Highpass 0.(	1000 Hz
University of Strathclyde	5-13 min	Closed	Two Biosemi system	64 Active	Left and right r	0.1-35 Hz	512 Hz
Istanbul Medipol University	2-11 min	Closed 4 mi Open 4 min	Brain Amp	32 Passive	A1 + A2 Electr	0.01-250 Hz	500 Hz
Takeda	7-8 min	Closed	Two Biosemi system	132 Active	Left and right r	unknown	1024 Hz

Supplementary Data 1. Details on fMRI and EEG data availability

Dataset	Country	Data obtained from	How to obtain individual data	MRI (1) fMRI(2) EEG(3)
ReDLat pros	Argentina, Chile, Colombia, Mexico, Peru	Agustín Ibañez	Contact agustin.ibanez@gbhi.org. An IRB approval and formal data sharing agreement is required.	(1) - (2)
Alzheimer's Disease Neuroimaging Initiative (ADNI)	USA	ADI - LONI	https://ida.loni.usc.edu/collaboration/access/appLicense.jsp	(1) - (2)
Centro de Gerociencia, Salud Mental y Metabolismo (GERO)	Chile	Andrea Slavchesky	Contact andrea.slachevsky@uchile.cl. An IRB approval and formal data sharing agreement is required.	(1) - (2)
Chinese Human Connectome Project (CHCP)	China	Science data bank	https://www.scidb.cn/en/detail?dataSetId=f512d085f3d3452a9b14689e9997ca94#p2	(1) - (2)
The frontotemporal lobar degeneration neuroimaging initiative (FTLDNI)	USA	ADI - LONI	https://ida.loni.usc.edu/collaboration/access/appLicense.jsp	(1) - (2)
ReDLat miller	USA	Bruce Miller - UCSF	Visit https://data.ucsf.edu/data-sharing or email datasharing@ucsf.edu	(1) - (2)
ReDLat pre	Argentina	Agustín Ibañez	Contact agustin.ibanez@gbhi.org. An IRB approval and formal data sharing agreement is required.	(1) - (2)
ReDLat pre	Peru	Nilton Custodio	Contact ncustodio@ipn.pe. An IRB approval and formal data sharing agreement is required.	(1) - (2)
ReDLat pre	Colombia	Diana Matallana	Contact dianamat@javeriana.edu.co. An IRB approval and formal data sharing agreement is required.	(1) - (2)
ReDLat pre	Colombia	Felipe Cardona	Contact felipe.cardona@correounivalle.edu.co. An IRB approval and formal data sharing agreement is required.	(1) - (2)
ReDLat pre	Mexico	Ana Luisa Sosa	Contact drasosa@hotmail.com. An IRB approval and formal data sharing agreement is required.	(1) - (2)
ReDLat pre	Chile	María Isabel Behrens	Contact behrensl@uchile.cl. An IRB approval and formal data sharing agreement is required.	(1) - (2)

ReDLat pre	Chile	Andrea Slavchesky	Contact andrea.slachevsky@uchile.cl. An IRB approval and formal data sharing agreement is required.	(1) - (2)
Japanese Strategic Research Program for the Promotion of Brain Science (SRPBS)	Japan	DecNef Project Brain Data Repository	https://bicr-resource.atr.jp/srpbsopen/	(1) - (2)
Centro de Neurociencias de Cuba	Cuba	Centro de Neurociencias de Cuba (CHBMP)	www.synapse.org/Synapse:syn22324937	(3) -
BrainLat	Argentina	Agustina Legaz	Contact alegaz@udesa.edu.ar	(3) -
BrainLat	Chile	Agustina Legaz	Contact alegaz@udesa.edu.ar	(3) -
Izmir University of Economics	Turkey	Gorsev Gener	Contact gorsev.yener@ieu.edu.tr	(3) -
Trinity College Dublin	Ireland	Francesca Farina	Contact francesca.farina@northwestern.edu	(3) -
Universidad de Antioquia	Colombia	Francisco Lopera	Contact floperar@gmail.com	(3) -
Universidad de Sao Paulo	Brazil	Mario Parra	Contact mario.parra-rodriguez@strath.ac.uk	(3) -
Aristotle University of Thessaloniki	Greece	Andreas Miltiadous	https://openneuro.org/datasets/ds004504/versions/1.0.5	(3) -
Universidad de Roma La Sapienza	Italy	Susana Lopez	Contact susanna.lopez@uniroma1.it	(3) -
University of Strathclyde	UK	Mario Parra	Contact mario.parra-rodriguez@strath.ac.uk	(3) -
Istanbul Medipol University	Turkey	Tuba Aktürk	Contact takturk@medipol.edu.tr	(3) -
Takeda	Chile	Daniela Olivares	Contact danielaolivaresvargas@gmail.com	(3) -
