# Supplementary material for: Brain clocks capture diversity and disparities in aging and dementia across geographically diverse populations
Source: Nat Med. 2024 Aug 26;30(12):3646–57. doi: 10.1038/s41591-024-03209-x (PMC11645278; doi:10.1038/s41591-024-03209-x)
Supplement: Supplementary file 2 — Reporting Summary [file 41591_2024_3209_MOESM2_ESM.pdf]

nature portfolio

Corresponding author(s): Agustin Ibanez

Last updated by author(s): Jul 18, 2024

Reporting Summary

Nature Portfolio wishes to improve the reproducibility of the work that we publish. This form provides structure for consistency and transparency in reporting. For further information on Nature Portfolio policies, see our Editorial Policies and the Editorial Policy Checklist.

Statistics

For all statistical analyses, confirm that the following items are present in the figure legend, table legend, main text, or Methods section.

n/a	Confirmed	
☐	☒	The exact sample size (*n*) for each experimental group/condition, given as a discrete number and unit of measurement
☐	☒	A statement on whether measurements were taken from distinct samples or whether the same sample was measured repeatedly
☐	☒	The statistical test(s) used AND whether they are one- or two-sided<br>*Only common tests should be described solely by name; describe more complex techniques in the Methods section.*
☐	☒	A description of all covariates tested
☐	☒	A description of any assumptions or corrections, such as tests of normality and adjustment for multiple comparisons
☐	☒	A full description of the statistical parameters including central tendency (e.g. means) or other basic estimates (e.g. regression coefficient) AND variation (e.g. standard deviation) or associated estimates of uncertainty (e.g. confidence intervals)
☐	☒	For null hypothesis testing, the test statistic (e.g. *F*, *t*, *r*) with confidence intervals, effect sizes, degrees of freedom and *P* value noted<br>*Give P values as exact values whenever suitable.*
☒	☐	For Bayesian analysis, information on the choice of priors and Markov chain Monte Carlo settings
☒	☐	For hierarchical and complex designs, identification of the appropriate level for tests and full reporting of outcomes
☐	☒	Estimates of effect sizes (e.g. Cohen's *d*, Pearson's *r*), indicating how they were calculated

Our web collection on statistics for biologists contains articles on many of the points above.

Software and code

Policy information about availability of computer code

Data collection	No software was used for data collection
Data analysis	The code used to preprocess and analyze the data of this work is available in an Open Science Foundation repository at the following address: https://osf.io/8zjf4/

For manuscripts utilizing custom algorithms or software that are central to the research but not yet described in published literature, software must be made available to editors and reviewers. We strongly encourage code deposition in a community repository (e.g. GitHub). See the Nature Portfolio guidelines for submitting code & software for further information.

Data

Policy information about availability of data

All manuscripts must include a data availability statement. This statement should provide the following information, where applicable:

- Accession codes, unique identifiers, or web links for publicly available datasets
- A description of any restrictions on data availability
- For clinical datasets or third party data, please ensure that the statement adheres to our policy

All preprocessed data are openly available at: https://osf.io/8zjf4/. The fMRI and EEG datasets comprise sources (a) currently publicly available for direct download after registration and access application, (b) available after contacting the researcher, or (c) accessible after IRB approval of formal data-sharing agreement in a process that can last up to 12 weeks. The fMRI sources that are publicly available for direct download are the following: Alzheimer's Disease Neuroimaging Initiative

(ADNI) (USA) (ida.loni.usc.edu/collaboration/access/appLicense.jsp), Chinese Human Connectome Project (CHCP) (China) (scidb.cn/en/detail?dataSetId=f512d085f3d3452a9b14689e9997ca94#p2), The frontotemporal lobar degeneration neuroimaging initiative (FTLDNI) (USA) (ida.loni.usc.edu/collaboration/access/appLicense.jsp), and Japanese Strategic Research Program for the Promotion of Brain Science (SRPBS) (Japan) (bicr-resource.atr.jp/srpbsopen/). The fMRI sources available after contacting the researcher include ReDLat USA by contacting Bruce Miller at UCSF through datasharing@ucsf.edu. The fMRI sources that require IRB approval and a formal data sharing agreement include: ReDLat pros (Argentina, Chile, Colombia, Mexico, Peru) by contacting Agustín Ibañez at agustin.ibanez@gbhi.org, Centro de Gerociencia Salud Mental y Metabolismo (GERO) (Chile) by contacting Andrea Slachevsky at andrea.slachevsky@uchile.cl, ReDLat pre (Argentina) by contacting Agustín Ibañez at agustin.ibanez@gbhi.org, ReDLat pre (Peru) by contacting Nilton Custodio at ncustodio@ipn.pe, ReDLat pre (Colombia) by contacting Diana Matallana at dianamat@javeriana.edu.co, ReDLat pre (Colombia -II) by contacting Felipe Cardona at felipe.cardona@correounivalle.edu.co, ReDLat pre (Mexico) by contacting Ana Luisa Sosa at drasosa@hotmail.com, ReDLat pre (Chile) by contacting María Isabel Behrens at behrensl@uchile.cl, and ReDLat pre (Chile) by contacting Andrea Slachevsky at andrea.slachevsky@uchile.cl. The EEG sources that are publicly available for direct download are Centro de Neurociencias de Cuba (CHBMP) (Cuba) (www.synapse.org/Synapse:syn22324937). The EEG sources that are available after contacting the researcher include BrainLat (Argentina) by contacting Agustina Legaz at alegaz@udesa.edu.ar, BrainLat (Chile) by contacting Agustina Legaz at alegaz@udesa.edu.ar, Izmir University of Economics (Turkey) by contacting Gorsev Gener at gorsev.yener@ieu.edu.tr, Trinity College Dublin (Ireland) by contacting Francesca Farina at francesca.farina@northwestern.edu, Universidad de Antioquia (Colombia) by contacting Francisco Lopera at floperar@gmail.com, Universidad de Sao Paulo (Brazil) by contacting Mario Parra at mario.parra-rodriguez@strath.ac.uk, Universidad de Roma La Sapienza (Italy) by contacting Susana Lopez at susanna.lopez@uniroma1.it, University of Strathclyde (UK) by contacting Mario Parra at mario.parra-rodriguez@strath.ac.uk, Istanbul Medipol University (Turkey) by contacting Tuba Aktürk at takturk@medipol.edu.tr, and Takeda (Chile) by contacting Daniela Olivares at danielaolivaresvargas@gmail.com. Indicators of air pollution, socioeconomic inequality (the Gini index), the burden of communicable, maternal, prenatal, and nutritional conditions, and the burden of non-communicable diseases were sourced from the updated country-specific data provided on the World Bank's platform (https://databank.worldbank.org/). Country-level gender inequality indexes (GII) are available on the World Health Organization's website (https://www.who.int/data/nutrition/nlis/info/gender-inequality-index-(gii)). For additional details, see Supplementary Data S1.

Research involving human participants, their data, or biological material

Policy information about studies with human participants or human data. See also policy information about sex, gender (identity/presentation), and sexual orientation and race, ethnicity and racism.

Reporting on sex and gender

Sex information was determined by self-report. No information regarding gender was inquired. We analyzed and reported sex differences between groups in brain age gaps. The total sample included 2970 female and 2336 male. Table 1 showed number of female and male in each subsample

Reporting on race, ethnicity, or other socially relevant groupings

No information regarding race or ethnicity was inquired. We did not considered race or ethnicity as a proxy of socioeconomic status.

Population characteristics

In this study, age, sex, and years of education covariates were considered for the human research participants. The full dataset included a total of 5306 participants, with 3509 healthy controls (HCs), 517 individuals with mild cognitive impairment (MCI), 828 individuals with Alzheimer's disease (AD), and 463 individuals with behavioral variant frontotemporal dementia (bvFTD).
For the fMRI dataset, the age of participants varied significantly across groups. Non-LAC healthy controls had an average age of 53.55 years (SD = 13.43), while LAC healthy controls had an average age of 65.34 years (SD = 11.44). Non-LAC individuals with MCI had an average age of 59.62 years (SD = 8.77), compared to 66.53 years (SD = 8.18) for their LAC counterparts. Non-LAC individuals with AD had an average age of 76.59 years (SD = 9.35), whereas LAC individuals with AD had an average age of 77.52 years (SD = 9.35). Non-LAC individuals with bvFTD had an average age of 73.14 years (SD = 8.56), while LAC individuals with bvFTD had an average age of 73.15 years (SD = 8.76).
Sex distribution also showed variability. Among non-LAC healthy controls, there were 470 females and 497 males, while LAC healthy controls included 261 females and 216 males. Non-LAC individuals with MCI had 114 females and 101 males, whereas LAC individuals with MCI had 84 females and 85 males. In the AD group, non-LAC individuals consisted of 112 females and 102 males, while LAC individuals included 262 females and 243 males. For bvFTD, non-LAC participants included 98 females and 92 males, and LAC participants included 105 females and 111 males.
Years of education also varied among participants. Non-LAC healthy controls had an average of 13.15 years of education (SD = 5.41), while LAC healthy controls had an average of 12.11 years (SD = 3.39). Non-LAC individuals with MCI had an average of 14.15 years (SD = 3.41), compared to 11.52 years (SD = 6.32) for LAC individuals with MCI. Non-LAC individuals with AD had an average of 13.12 years (SD = 5.34), whereas LAC individuals with AD had an average of 8.89 years (SD = 4.34). Non-LAC individuals with bvFTD had an average of 11.16 years (SD = 3.56), while LAC individuals with bvFTD had an average of 7.89 years (SD = 3.36).
For the EEG dataset, non-LAC healthy controls had an average age of 58.98 years (SD = 12.03), while LAC healthy controls had an average age of 66.74 years (SD = 13.94). Sex distribution in the EEG dataset showed that non-LAC healthy controls consisted of 470 females and 99 males. In the LAC healthy controls, there were 954 females and 532 males, with 111 females and 22 males among LAC individuals with MCI, 85 females and 23 males among LAC individuals with AD, and 39 females and 18 males among LAC individuals with bvFTD.
Years of education for the EEG dataset also showed differences. Non-LAC healthy controls had an average of 14.85 years (SD = 4.91). LAC healthy controls had an average of 13.92 years (SD = 3.39), individuals with MCI had an average of 8.12 years (SD = 4.34), individuals with AD had an average of 10.75 years (SD = 6.32), and individuals with bvFTD had an average of 14.38 years (SD = 5.49).

Recruitment

Participants were selected following a stratified design in each country

Ethics oversight

The respective IRB of each institution that contributed EEGs and/or fMRIs to this study approved the acquisitions and protocols, and all the participants signed a consent form following the declaration of Helsinki.

Note that full information on the approval of the study protocol must also be provided in the manuscript.

Field-specific reporting

Please select the one below that is the best fit for your research. If you are not sure, read the appropriate sections before making your selection.

☒ Life sciences ☐ Behavioural & social sciences ☐ Ecological, evolutionary & environmental sciences

For a reference copy of the document with all sections, see nature.com/documents/nr-reporting-summary-flat.pdf

Life sciences study design

All studies must disclose on these points even when the disclosure is negative.

Sample size	The sample sizes for this study were determined using a data-driven approach to ensure the effectiveness of deep learning regressors in predicting brain age gaps. Preliminary analyses informed by pilot data and previous studies guided the decision to include a total of 5306 participants. This large sample size, encompassing 3509 healthy controls, 517 individuals with mild cognitive impairment, 828 individuals with Alzheimer's disease, and 463 individuals with behavioral variant frontotemporal dementia, provides a comprehensive dataset. This dataset captures the necessary variability for reliable model training and validation, enhancing the generalizability and predictive performance of the deep learning models. By leveraging such a sizable and diverse cohort, the study aims to develop models that can accurately predict brain age gaps across different populations and conditions, thereby ensuring robust and reliable outcomes.
Data exclusions	No data was excluded
Replication	The models were tested in six independent out of sample datasets. All attempts at replication were successful.
Randomization	Subsamples matched by age, sex, and education to the healthy control group.
Blinding	The investigators were blinded to group allocation during data collection and analysis.

Reporting for specific materials, systems and methods

We require information from authors about some types of materials, experimental systems and methods used in many studies. Here, indicate whether each material, system or method listed is relevant to your study. If you are not sure if a list item applies to your research, read the appropriate section before selecting a response.

Materials & experimental systems

n/a	Involved in the study
☒	☐ Antibodies
☒	☐ Eukaryotic cell lines
☒	☐ Palaeontology and archaeology
☒	☐ Animals and other organisms
☒	☐ Clinical data
☒	☐ Dual use research of concern
☒	☐ Plants

Methods

n/a	Involved in the study
☒	☐ ChIP-seq
☒	☐ Flow cytometry
☒	☐ MRI-based neuroimaging

Plants

Seed stocks	*Report on the source of all seed stocks or other plant material used. If applicable, state the seed stock centre and catalogue number. If plant specimens were collected from the field, describe the collection location, date and sampling procedures.*
Novel plant genotypes	*Describe the methods by which all novel plant genotypes were produced. This includes those generated by transgenic approaches, gene editing, chemical/radiation-based mutagenesis and hybridization. For transgenic lines, describe the transformation method, the number of independent lines analyzed and the generation upon which experiments were performed. For gene-edited lines, describe the editor used, the endogenous sequence targeted for editing, the targeting guide RNA sequence (if applicable) and how the editor was applied.*
Authentication	*Describe any authentication procedures for each seed stock used or novel genotype generated. Describe any experiments used to assess the effect of a mutation and, where applicable, how potential secondary effects (e.g. second site T-DNA insertions, mosiacism, off-target gene editing) were examined.*
